# Network structure of depression symptomology in participants with and without depressive disorder: the population-based Health 2000–2011 study

**DOI:** 10.1007/s00127-020-01843-7

**Published:** 2020-02-11

**Authors:** Christian Hakulinen, Eiko I. Fried, Laura Pulkki-Råback, Marianna Virtanen, Jaana Suvisaari, Marko Elovainio

**Affiliations:** 1 Department of Psychology and Logopedics, Faculty of Medicine, University of Helsinki, P.O. Box 9, 00014 Helsinki, Finland; 2 The Finnish Institute for Health and Welfare, Helsinki, Finland; 3 Department of Clinical Psychology, Leiden University, Leiden, The Netherlands; 4 School of Educational Sciences and Psychology, University of Eastern Finland, Joensuu, Finland

**Keywords:** Network, Connectivity, Depression, Symptoms

## Abstract

**Purpose:**

Putative causal relations among depressive symptoms in forms of network structures have been of recent interest, with prior studies suggesting that high connectivity of the symptom network may drive the disease process. We examined in detail the network structure of depressive symptoms among participants with and without depressive disorders (DD; consisting of major depressive disorder (MDD) and dysthymia) at two time points.

**Methods:**

Participants were from the nationally representative Health 2000 and Health 2011 surveys. In 2000 and 2011, there were 5998 healthy participants (DD−) and 595 participants with DD diagnosis (DD+). Depressive symptoms were measured using the 13-item version of the Beck Depression Inventory (BDI). Fused Graphical Lasso was used to estimate network structures, and mixed graphical models were used to assess network connectivity and symptom centrality. Network community structure was examined using the walktrap-algorithm and minimum spanning trees (MST). Symptom centrality was evaluated with expected influence and participation coefficients.

**Results:**

Overall connectivity did not differ between networks from participants with and without DD, but more simple community structure was observed among those with DD compared to those without DD. Exploratory analyses revealed small differences between the samples in the order of one centrality estimate participation coefficient.

**Conclusions:**

Community structure, but not overall connectivity of the symptom network, may be different for people with DD compared to people without DD. This difference may be of importance when estimating the overall connectivity differences between groups with and without mental disorders.

**Electronic supplementary material:**

The online version of this article (10.1007/s00127-020-01843-7) contains supplementary material, which is available to authorized users.

## Introduction

Depressive disorders (DD), including major depressive disorder (MDD) and dysthymia, are highly prevalent mental disorders with high comorbidity with other mental disorders. Although they have been under systematic investigation for decades, depressive disorders remain poorly understood, and treatment efficacy has been modest [1]. It has been traditionally assumed that depressive symptoms arise from common pathogenic pathways. Recently, this common cause-approach has been challenged [2–4] by research showing that different depressive symptoms are associated with different risk factors [5], different patterns of comorbidity [6], and are associated with different levels of impairment [3]. Consistent with the above evidence of differential relations between symptoms and varying outcomes, depression symptomology has been conceptualized as a dynamic network, suggesting that depressive disorders are an emergent property that derives from mutual interactions among symptoms in a causal system [7]. The model assumes that depression is a complex dynamic system where individuals suffering from depression have a different architecture of symptom relations than those who experience depressive symptoms but have not passed the threshold of clinical diagnosis. The architecture of symptoms that characterizes those with a high risk of depression may form an emergent state: ‘depression’. Such a state can be sustained via vicious circles, and can be difficult to escape [8].

This network theory of depression is grounded in theories in clinical psychology. For instance, cognitive behavioral therapy focuses on negative feedback loops potentially leading to more severe emotional problems [9]. Although the network approach has generated much interest [4, 8, 10–12], numerous questions have remained open. We introduce three especially relevant topics below. First, one of the important features which are discussed as potentially differentiating the symptom networks of depressed people and others is connectivity [7], i.e. the amount and strength of relations among symptoms. People more vulnerable to develop depression have been suggested to have a denser symptom network and overall stronger ties between symptoms than those who are less vulnerable. In clinical samples, this may mean that more densely connected networks in patients with MDD would also predict less probable recovery [8]. However, the literature on the topic is very limited, and empirical evidence is mixed: one previous study has supported this notion [13], and a second one has not [14].

Second, many network studies have examined what symptoms are the most central (i.e. interconnected) in MDD symptom networks, because such symptoms have been speculated to be promising targets for intervention [10]. Most of the studies so far have used clinical samples [12, 15–17], and few have used community samples analyzing also the sub-threshold symptoms [10, 18]. Interestingly, results are mixed, and do not seem to replicate well across studies. For example, whereas in a large clinical sample, Fried et al. [12] found that sad mood and energy loss were the most central symptoms, a time-series study conducted by Bringmann and colleagues [11] concluded that loss of pleasure was the most central symptom. Contrary to these findings, in a sample of 5952 Han Chinese women with recurrent MDD, psychomotor changes, hopelessness and decreased self-confidence were found to be the most central symptoms and among the least central was loss of interest [16]. Jones and others [17] concluded that concentration impairment, sadness, and fatigue were the most central nodes among individuals with obsessive–compulsive disorder with comorbid depression. These differences might be explained by variability in the samples, designs, and depression inventories used [12].

In addition, of the three most widely used centrality measures, i.e., closeness, betweenness and node strength [19], closeness and betweenness have suggested to be difficult to interpret in psychological networks, because they are based on assumption that do not hold when studying association between variables [20]. The most suitable centrality measure may thus be strength centrality that measures the weighted number of connections of a focal node and thereby the degree to which it is involved in the network. Moreover, “expected influence” indices, which distinguish between positive and negative edges, may be more suitable for evaluating centrality in networks with various community structures [21]. Similarly, indicators, such as participation coefficient, that measures the strength of a node’s connections within its community may be useful when comparing symptoms networks with different community structures. It has also repeatedly been shown that measures detecting depression severity often fail to show uni-dimensionality or measurement invariance over time [4], which makes cliques highly likely in depression data, given the mathematical equivalence between factor and network models [22]. Furthermore, the most commonly used centrality measures are often calculated without considering the effects that the potential differences in the local systemic entities, such as community structures within the networks [23], have on centrality measures. This could have contributed to the inconsistent results of centrality estimates across publications in the literature.

**Fig. 1:**
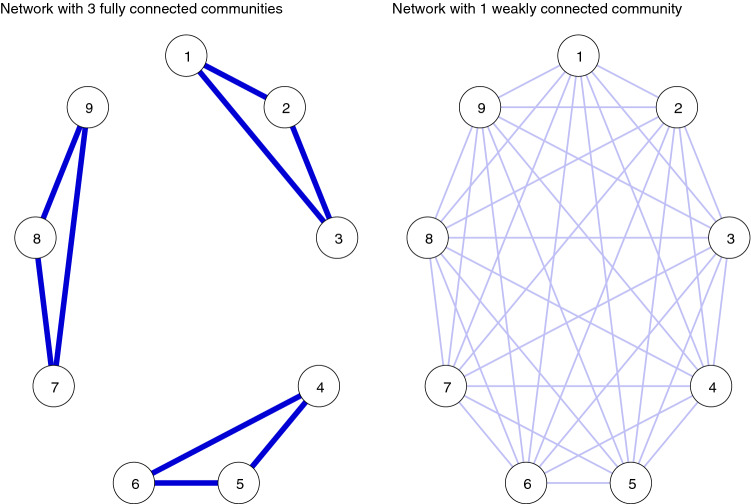
Illustration how different community structures can lead to the same connectivity and centrality measures

Third, many previous studies have estimated depressive symptoms (and also other symptom networks) without considering the fact that symptom networks may include many locally connected structures, referred to as communities or cliques. To the best of our knowledge, so far only few studies have investigated the community structure of depressive symptoms networks [16, 24]. This is a gap in the literature, as connectivity is a global measure: different community structures can lead to the same connectivity; for illustration of this effect, see Fig. 1. On the left, there is a network of nine nodes with three nodes structured in three communities that are fully and strongly connected; all edge weights are 0.99; however, there are no edges from any community to any other. The overall connectivity of 9 present and 27 absent edges is 9. On the right-hand side is a fully connected network with 9 edges but only one community, with much smaller edge weights of 0.25. This network has the same overall connectivity as network 1 (9), although the architecture and the conclusions we potentially make of the connections between nodes are probably different (for details of the example, see Online Supplement Appendix).

In sum, although the number of studies analyzing depressive symptom networks have increased rapidly, some crucial question remains open. First of all, results are conflicted to whether individuals with depression have higher or lesser symptom connectivity, or whether high connectivity is actually “good or bad” [8]. Second, it remains unclear which of the symptoms are more central than others in the depression symptom network. Third, it is also not clear whether there are differences in the community structures in the symptom networks between individuals with and without depression, and whether these differences affect the conclusions about the connectivity and centrality of individual symptoms.

To address these open questions, the present study examined self-reported depressive symptom networks using data from the nationally representative Health 2000–2011 surveys in Finland. The specific aims were to examine whether there were differences between those with depressive disorder (DD) and those without (A) in overall connectivity in symptom networks, (B) in centrality of the symptoms, and (C) in community structures of the symptom networks using metrics taking into account the community structure and local connectivity (expected influence step 2 and participation coefficients) to find out whether there are differences between the groups that network theory has traditionally not analyzed.

## Methods

### Sample

The data were derived from two data collection phases of the multidisciplinary epidemiological survey, “The Health 2000–2011”, which was carried out in Finland in 2000–2001 and in 2010–2011. As described in detail elsewhere [25], in 2000, a nationally representative sample was drawn among adults aged 30 years or over and living in the mainland of Finland. Two-stage clustered sampling of 15 largest towns and 65 health districts in Finland was used and individuals over 80 years were oversampled (2:1). In addition, young adults’ sample of individuals who were between 18 and 29 years old were collected using shortened version of the study protocol. In 2011, all participants who were alive, living in Finland, and had not refused to participate, were invited to take part of new data collections wave [26]. In addition, participants from the young adults’ sample of Health 2000, were included.

In Health 2000, a total of 7419 participants (93% of the 7977 subjects alive at the first day of the first phase of the survey) participated to one or more phase of the study. Of these, 6354 participated in the clinical examination, which included, e.g., the Composite International Diagnostic Interview (CIDI), which was reliably performed for 6005 participants (75% of the original sample). In Health 2011, a total 6740 participants (67% of those who were invited) participated at least one to one phase of the study. Of these participants, 4729 participated in the health examination.

The present study was restricted to those participants who had participated in CIDI and responded to BDI-13 questionnaire in 2000 and/or 2011. This resulted to a total of 5998 participants without depressive disorder (DD) and 595 with depressive disorder. Participants with other mental disorders in 2000 or in 2011 were excluded.

## Measures

### Diagnoses

Depression diagnoses were based on the Finnish translation of the Munich version of the Composite International Diagnostic Interview (M-CIDI, [27]. CIDI uses operationalised criteria for DSM-IV diagnoses and allows an estimation of DSM-IV diagnoses for mental disorders. In the present study, a computerized version of CIDI was used [27]. The translation of the CIDI-items into Finnish was based on the original English items of CIDI and was made pairwise by psychiatric professionals. The process included consensus meetings, expert opinions, an authorized translator’s review, and pilot testing with both informed test participants and unselected real participants.

The CIDI interview has been found to be a valid and reliable instrument [28, 29]. The interviews were performed to determine the 12-month prevalence of depressive (dysthymia or major depressive disorder, MDD), anxiety and alcohol use disorders. The interviewers were non-psychiatric health professionals who were trained in conducting CIDI interviews. Trainers were psychiatrists or physicians trained by a WHO authorized trainer. The Kappa values for the two interviews were 0.88 (95% CI 0.64–1.0, observer agreement 94%) for major depressive disorder, and 0.88 (95% CI 0.64–1.0, observer agreement 98%) for dysthymia [30]. In depressive disorders, the CIDI interview differentiates also between dysthymic disorder and MDD. Furthermore, the most recent timing (or appearance) of each symptom was also recorded (time frame of depression), allowing for estimates about when the diagnostic criteria were fulfilled most recently. In the current study, the variable for psychiatric diagnosis was coded as DD (includes MDD with or without dysthymia and dysthymia) and no DD (or other mental disorders).

### Depressive symptoms

**Fig. 2:**
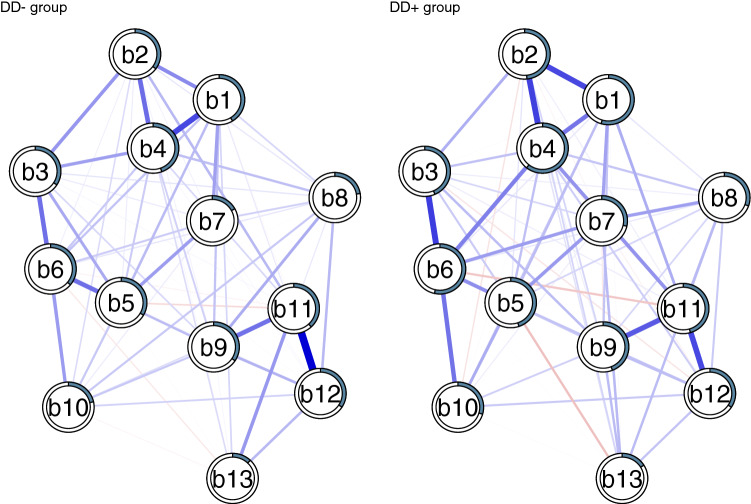
Visualization of the Fused Graphical Lasso (FGL) estimated networks of depressive symptoms in participants without (DD−) and with (DD+) major depressive disorder or dysthymia. Symptoms are as follows: b1 = Depressed mood/sadness; b2 = Pessimistic about the future; b3 = Low self-esteem/past failure; b4 = Loss of pleasure/dissatisfaction; b5 = Feeling guilty; b6 = Feeling disappointed in oneself/self-dislike; b7 = Self harm; b8 = Loosing interest in other people; b9 = Difficulties in decision-making; b10 = Dissatisfaction with once appearance/worthlessness; b11 = Loss of energy; b12 = Tiredness; b13 = Loss of appetite

Depressive symptoms were assessed using the Beck Depression Inventory (BDI) [31]. In 2000 21-item version was used, and in 2011 the 13-item version [32]. In the current study, we used those 13 items of BDI that were measured at both time points (Fig. 2).

### Statistical methods

We estimated network models, community structures and graph-theoretical measures in multiple steps. All statistical analyses and used statistical packages are explained in detail in the online supplement. First, we estimated network structures of depression symptoms in two sub-groups based on CIDI: (1) individuals without a diagnosis of DD (DD−) and (2) individuals diagnosed with DD or dysthymia (DD+). The network structures were analyzed using the responses in 2000 or 2011 (if they responded only once) and means of their responses (if they responded in 2000 and in 2011) for each individual. To obtain networks for non-depressed and depressed groups, we estimated polychoric correlations among symptoms (for robustness analyses with Spearman correlations, see the online supplement), and then estimated the depression symptom network using the Fused Graphical Lasso (FGL).

Second, we assessed the predictability of the individual symptoms (how much of the variance of each symptom is explained by the other nodes in the network) using Mixed Graphical Models. After calculating the predictability results, we included these parameters into the FGL networks. We used the R package “qgraph” [33] to plot the networks. Third, we compared the connectivity of the networks between DD− and DD+ groups using the “NetworkComparisonTest” (NCT) R-package [34]. Fourth, we evaluated the community structure of the symptom networks in both groups using the walktrap-algorithm [35] and “igraph”-package [36] (for robustness analyses via the spinglass algorithm, see the online supplement). Sub-network structures of depressive symptoms in different groups were tested using the minimum spanning trees (MST) [37].

Fifth, we calculated node strength, which was our primary centrality measure, and also estimated “Expected influence” centrality index as well as participation coefficient for each node. Correlations between strength centrality measures and expected influence were calculated to evaluate the overall similarity between the groups. Sixth, we tested the parameter accuracy of edges and centrality estimates in the symptom networks, using the R package “bootnet”, via a bootstrap sampling procedure with 1000 iterations. We evaluated the stability of the strength centrality metrics using the correlation stability (CS) coefficient by repeatedly correlating centrality metrics of the original data set with those calculated from subsamples including progressively fewer participants. The CS-coefficient represents the maximum proportion of participants that can be dropped while maintaining 95% probability that the correlation between centrality metrics from the full data set and the subset data are at least 0.7, and should be above 0.5

As additional sensitivity analyses, we bootstrapped centrality scores (1000 samples) to estimate the uncertainty in the correlation between the centrality scores of the DD− and DD+ group and examined the community structures in more detail (for details see the online supplementary appendix).

All analyses were conducted using R 3.5.1 (R Core Team 2018).

## Results

### Descriptive statistics

There were 5998 DD− participants and 595 DD+ participants with data for either or both measurement points. Differences between the average symptom level over time points were all significant between DD− and DD+ groups, the mean of all symptoms in DD− group was 0.19 and in DD+ group 0.55 (difference = − 0.35, 95% CI [− 0.40, − 0.33]). The greatest differences were found for sadness (means 0.15/0.72) and guilty feelings (means 0.22/0.72), and the smallest difference was found for change in appetite (means 0.07/0.22) and in self-dislike (means 0.08/0.36). The means and standard deviations and zero-order correlation matrices of individual symptoms are presented in the Online Supplement (Supplement Figs. 1 and 2). The Spearman correlations between the symptom profiles was 0.80 suggesting rather strong similarities across MDD groups.

### Network structure

The visualization of the FGL networks for DD− and DD+ groups are presented in Fig. 2. The predictability (amount of explained variance of each symptoms by all the other symptoms) is illustrated by the percentage of shaded area in the pie. Depressive symptoms descriptively explained a larger proportion of the variance of the other symptoms in DD+ (mean explained variance 41%) than in DD+ participants (mean explained variance 31%). This finding translates into somewhat stronger associations between symptoms in participants with MDD than in those without (average edge weight 0.07 in the DD+ group vs 0.06 in the DD− group). The internal consistency (Cronbach’s alpha) was also slightly higher in DD+ (0.89) than in DD− (0.84) group. When comparing the networks across groups, the Network Comparison Test revealed no significant differences regarding network structures (*M* = 0.12; *p* = 0.77) or network connectivity (global strength) (difference 0.08, *p* = 0.87), with connectivity estimates of 5.4 for DD+ group and 5.5 for DD− group. Overall similarity was evaluated by calculating the correlations between the edge weights across networks for each pair of networks (Supplement Figs. 3 and 4). Spearman correlation was 0.65, also indicating rather strong similarity.

**Fig. 3:**
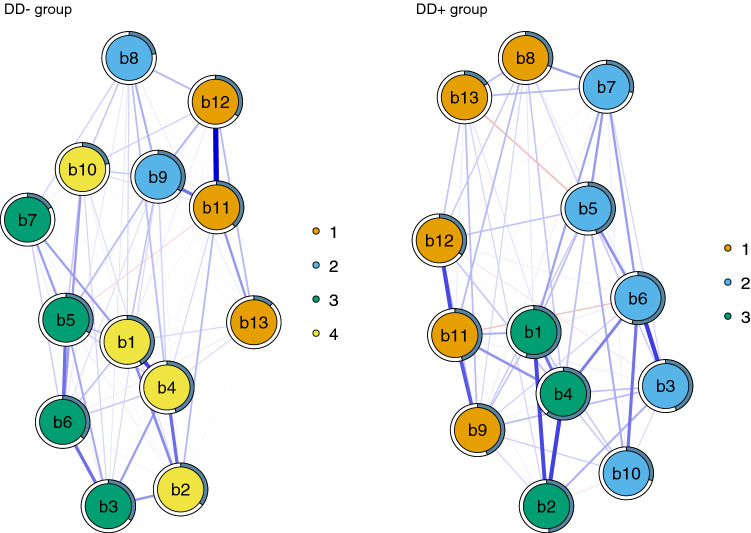
The community structure of the networks of depressive symptoms in DD− and DD+ participants

**Fig. 4:**
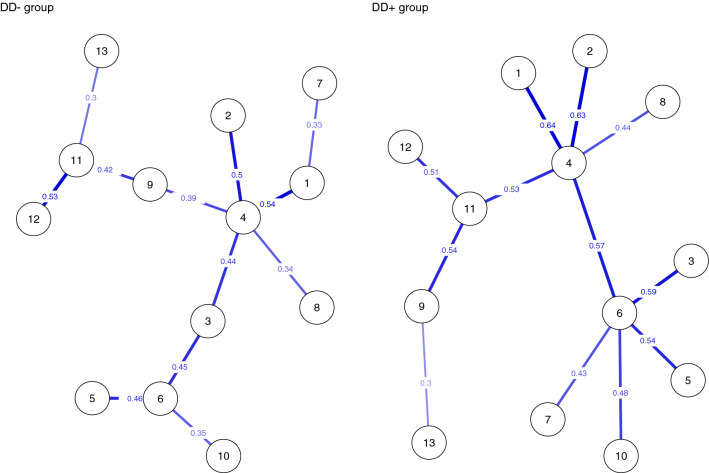
Minimum spanning trees of depressive symptoms (between individual networks) in DD− and DD+ participants

All else being equal, we identified some differences in the community structures of the networks between MDD groups (Fig. 3). In DD− group, the walktrap-algorithm suggested four different communities, but only three communities were suggested in DD+ group (results remained the same when rerunning the algorithm ten times with random seeds). Minimum spanning trees supported the less uniform structure of symptoms in DD− group compared to DD+ group although the most central nodes were partly the same in both groups (Fig. 4). The nodes closer to the center of the tree (i.e. nodes that feature more edges) are most central. Loss of pleasure, past failure, and indecisiveness were the most central symptoms in DD− and in DD+ group they were loss of pleasure, self-dislike, and loss of energy.

**Fig. 5:**
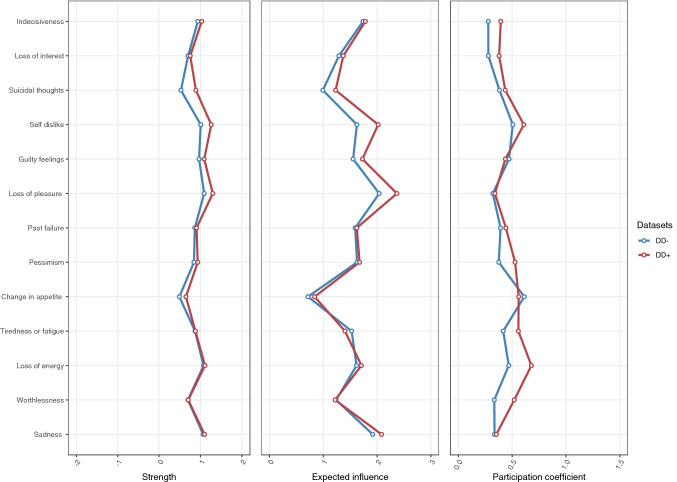
The two centrality measures: node strength(unstandardized) and expected influence and participation coefficient for depressive symptoms in DD− and DD+

The centrality estimates (node strength and expected influence) and participation coefficients are shown in Fig. 5. Loss of pleasure, sadness, loss of energy, and self-dislike had the greatest node centrality strength in DD+ and in DD− group and they were also more central than 50% or more of other nodes in the network (bootstrapped difference tests for strength centrality are presented in Supplement Figs. 5–8). The strength centrality profiles were very similar in DD− and DD+ groups, with a correlation of 0.85 suggesting strong similarity across groups. The expected influence profiles were also similar (*r* = 0.89) and again especially loss of pleasure, self-dislike, and sadness were high, all but even higher in DD+ group. The participation coefficients suggested that the loss of energy and self-dislike were central symptoms in DD+ group (Fig. 4), with a correlation of *r* = 0.67 across groups. The CS coefficients indicated a stable order of strength centrality estimation, with values of 0.67 (DD+) and 0.75 (DD−).

Sensitivity analyses regarding the centrality indexes confirmed our findings reported above (see online supplement for details). Sensitivity analyses related to community structure showed that the community structure in those participants with DD+ were relatively stable and three-community-solution was the most common (Supplement Fig. 9). Among the DD− participants, network was clearly less stable (Supplement Fig. 10).

## Discussion

The present study examined depressive symptom networks using data from a nationally representative general population sample. Results showed that there were no differences in the overall connectivity of symptom networks between participants with and without DD (major depressive disorder and dysthymia). Whereas simpler community structure was observed among those participants with DD, the differences in centrality measures between participants with and without DD were relatively small.

Our findings regarding the overall network connectivity were somewhat unexpected and not consistent with all prior work. Specifically, some studies showed an increased network connectivity in participants with depression [38, 39]. The network theory—supported by prior studies using both empirical and simulated data—has suggested that network connectivity may be a key feature leading to attractor states with large number of active symptoms and thus to clinical depression [8]. Strong connections between symptoms indicate that symptoms more easily affect each other and thus maintain and trigger negative systemic states. Our findings suggesting that there were no differences in connectivity between groups of people with and without DD, do not provide strong support for the inferences of the theory. However, these findings are in line with a intervention study, where stronger symptom connectivity was not associated with treatment prognosis [14]. They also are in line with another study where the connectivity of depressive symptoms was found to increase during antidepressant treatment in a very large clinical trial (the STAR*D study) while the overall severity of depression decreased [4].

In the present study, fewer communities, and thus simpler community structure was found in those participants with DD. This was unexpected given previous work that found that decreases in depressive symptoms across time were associated with structures that became less multifactorial (i.e. increasingly more unidimensional) [4]. Especially our finding that the community structure was less stable among participants without depression (see online supplement for more details) warrants further investigation.

The most central symptoms in the depressive symptoms network of the participants with DD were (a) loss of pleasure, (b) self-dislike, (c) sadness and (d) loss of energy. In the present study, some less frequently used centrality measures, which took more efficiently into account the community structure, were used. However, only minor differences compared to the strength centrality measures (correlation range from 0.59 to 0.89) were found. Based on all indicators used in this study, loss of energy, and loss of pleasure were consistently central in those with DD and the differences between those without and with DD were relatively small. Similar results have been previously reported, suggesting that sadness [12] or loss of pleasure (Bringmann et al. 2015) would be the most central symptoms in MDD. However, other studies have found different symptoms to be most central [16, 17], indicating that central symptoms might differ across samples. It is also important to notice that symptoms of sadness and/or anhedonia were required for depression diagnosis in this sample, which may bias centrality statistics.

Although it is tempting to assume that the most central symptoms also have a strong causal role in the network, empirical investigations into the matter are scarce. Rodebaugh and co-workers [40] examined whether central symptoms in a network constructed using a cross-sectional data predicted the correlation between change in a given node using the same data and change in other symptoms across treatment also in another dataset. They found that centrality predicted which nodes were more strongly associated with change above and beyond other predictors, but that prediction was restricted to that specific network and data where the centrality was determined. Thus, the higher centrality was associated with a stronger association with change across the entire symptom network, but only among those specific symptoms where the centrality measures were detected. There are multiple problems in interpreting central symptoms as the most influential, (the most central may be just the end point or just the one with the greatest variability, see: https://psych-networks.com/how-to-not-interpret-centrality-values-in-network-structures/) and recently the whole basis of measuring centrality in psychological networks that do not have similar features (serial flow of connections) as social networks, has been challenged [20]. In the present study, we tried to overcome some of these problems using centrality measures that are not based on shortest path measures (strength centrality) and by taking into account the community structure within the network (participation coefficient) [41].

Recently, some work has criticized the application of centrality metrics derived from social network analysis to psychological data [20]. This may be especially problematic if centrality measures are considered—as they often seem to be—as measures of symptom importance. These metrics assume that there are no qualitative differences between nodes, which is a contentious assumption. In psychological networks, especially symptom networks, it is difficult to interpret that suicidal thoughts would be as important as changes in appetite and thus, focusing only on the connections in psychological networks to find the most central node would be problematic. It is also possible that the observed differences in central symptoms between groups may be a result of sampling variability changing the absolute rank order of symptoms without there necessarily being any differences in centrality of the symptoms [42]. Given that prior research was in part based on small samples and lacked investigations whether the most central symptoms was substantially or significantly more central than other symptoms (e.g. via the centrality difference test [43]), this raises doubts as to how meaningful differences in reported centrality differences in the literature are, and we hope the at least in part large sample size of the present study adds to the literature in that regard.

### Strengths and limitations

Main strengths of the current study are a population-based sample, which is a representative of Finnish general adult population, and the use of CIDI to identify participants with DD during the last 12 months. Some limitations need to be taken into account when current findings are interpreted. The original sample of the Finnish Health 2000 survey included 8028 subjects of whom 6005 (75%) were interviewed with the CIDI. It has been shown participants who did not participate had more depressive symptoms than those who participated, indicating that they were more likely to suffer from DD. However, the aim of the current study was not estimate the prevalence of DD, and CIDI has been found to have acceptable psychometric properties [44]. Second, cohort effects could bias or confound our results, although we do not think this is highly likely, because there were no differences in the levels of depressive symptoms or DD prevalence between the two time points [45]. Third, the analytical design was cross-sectional, preventing us making any inferences about the direction of the associations or development the network structures. For example, participants who were not diagnosed with depression could be in remission. Fourth, we used regularized models which make groups with different sample sizes difficult to compare (see supplement analyses for analyses in which we subsampled participants to obtain equal sample sizes). Fifth, we mainly relied on community detection results based on the walktrap-algorithm (see supplement analyses for results based on the spinglass algorithm). Six, from the all possible depressive symptoms, our investigation is limited to those which are included in BDI-13, and thus other important symptoms may be missing. Finally, although depression diagnosis was based on structured interview (M-CIDI), and not on BDI scores, Berkson's bias could potentially influence our results [46].

## Conclusions

To conclude, we found that community structure, but not overall connectivity or symptom centrality, of the symptom network may be different between participants with and without DD. This difference could be important when estimating the overall connectivity differences in symptoms between groups with and without mental disorders.

## Electronic supplementary material

Below is the link to the electronic supplementary material.

Supplementary file1 (DOCX 4346 kb)

## Data Availability

Authors do not have permission to share the research data. Data access can be applied from the Finnish institute for health and welfare. For details, see: https://thl.fi/en/web/thlfi-en/research-and-expertwork/projects-and-programmes/health-2000-2011/information-for-researchers. Code for the statistical analyses is available from https://github.com/Melovainio/Network_depression.
