# Supplementary material for: Network structure of depression symptomology in participants with and without depressive disorder: the population-based Health 2000–2011 study
Source: Soc Psychiatry Psychiatr Epidemiol. 2020 Feb 11;55(10):1273–82. doi: 10.1007/s00127-020-01843-7 (PMC7544719; doi:10.1007/s00127-020-01843-7)
Supplement: Supplementary file 1 — Supplementary file1 (DOCX 4346 kb) [file 127_2020_1843_MOESM1_ESM.docx]

**ONLINE SUPPLEMENT APPENDIX**

**Supplement Text.** R-code for Figure 1 analyses

**Supplement Text.** Additional details of statistical analyses

**Supplement Text.** Results from the sensitivity analyses

**Supplement Figure 1.** Means and SDs of the original BDI -13 – items in those without and with MDD (SDs scaled to below 1)

**Supplement Figure 2.** Correlations coefficients of depressive symptoms in 2000 for participants with and without depressive disorder

**Supplement Figure 3.** Edge weights and Cis

**Supplement Figure 4.** Centrality stability

**Supplement Figure 5:** Edge weight difference test in DD- group

**Supplement Figure 6:** Edge weight difference test in DD+ group

**Supplement Figure 7:** Centrality difference test in DD- group

**Supplement Figure 8:** Centrality difference test in DD+ group

**Supplement Figure 9.** Community structures for the two most frequently occurring solutions in DD+ network

**Supplement Figure 10.** Community structures for the two most frequently occurring solutions in DD- network

**Supplement Figure 11.** Heatmap for the DD+ network

**Supplement Figure 12.** Heatmap for the DD- network

**R-code for Figure 1 analyses**

# 3 cliques

net <- matrix(0,9,9)

net[1:3,1:3] <- .99999

net[4:6,4:6] <- .99999

net[7:9,7:9] <- .99999

diag(net) <- 0

sum(net)/2 # connectivity ~ 9

# 1 clique

net1 <- matrix(0,9,9)

net1[1:9,1:9] <- .25

diag(net1) <- 0

sum(net1)/2 # connectivity ~ 9

# plot

library(qgraph)

layout(t(1:2))

nw <- qgraph(net, details=F, title="Network with 3 fully connected communities")

nw1 <- qgraph(net1, layout=nw$layout, details=F, maximum=1, title="Network with 1 weakly connected community")

**Supplement Text.** Additional details of statistical analyses

Fused Graphical Lasso (FGL), which was used to estimate the depression symptom network, extends the graphical least absolute shrinkage and selection operator (gLasso) by applying a penalty not only to the sum of the absolute values of the elements of the concentration matrix multiplied by the tuning parameter λ_1_, but also to the sum of the absolute values of the differences between the corresponding elements of the concentration matrix across groups, multiplied by another tuning parameter (λ_2_). This method requires setting two tuning parameters: one (λ_1_) is analogous to the tuning parameter in the graphical lasso and regulates sparsity. The other (λ_2_) affects the similarity of the networks estimates in different groups: The higher λ_2_, the more similar the resulting networks will be [1]. As for the graphical lasso, the values of both tuning parameters can be selected using information criteria or a cross-validation approach (we used cross-validation). The FGL, coupled with tuning parameters selection via information criteria or cross-validation, has some advantages over independent network estimates in different groups. The FGL identifies which edges may be identical, and which may be different in the different groups without worsening the model fit. The idea behind considering edges as equal or different is the same that is used in the graphical lasso methodology to identify edges that can be shrunk to zero without compromising model fit, therefore this method provides a good solution to the issue of network comparison [2]. This method makes the networks computed in different groups often more parsimonious, since they involve less unique parameters. The FGL improves network estimates by exploiting similarities among different groups: If two groups have many elements in common, estimating such elements in both groups improves the estimates. When exploiting similarities does not improve model fit, the tuning parameter selection procedure picks a value of the λ_2_ parameter that is very close to zero or zero. In this case, no penalty is imposed to the differences among groups and the FGL reduces to estimating two separate Gaussian Graphical Models, albeit with a shared sparsity parameter. Thus, true differences among groups are not masked. We also calculated correlations between the edge weights across networks to evaluate the overall similarity. FGL has been used, for instance, for comparing networks between borderline personality disorder patients and a community sample [3].

Mixed Graphical Models (MGMs) were estimated via 1-regularized (Lasso) neighborhood regression, the neighborhood of a node being the set of nodes connected to it. R package “mgm”[4] was used for the analyses.

“NetworkComparisonTest” (NCT) [5] was used to compare the connectivity of the networks between DD- and DD+ groups. The NCT is a two-tailed permutation test involving the repeated (100,000 times) calculation of the difference between two groups of randomly regrouped individuals. It results in a distribution under the null hypothesis (on the assumption that both groups are equal), which can be used to test the significance of any difference between the groups. The standard NCT provides also the measure for overall connectivity in different networks. We tested the overall connectivity using the NCT-bootnet -function that uses polychoric correlations. We also calculated the correlations between the edge weights across networks to obtain an overall coefficient of network similarity,

The walktrap-algorithm [6] was used to evaluate the community structure of the symptom networks in DD- and DD+ groups. The walktrap -algorithm begins with a random search from each node to surrounding nodes to satisfy a proportion of high internal edges to surrounding nodes (many, dense connections to surrounding nodes) compared to the proportion of edges between the node and more distant nodes (few, distant connections to more remote nodes). The proportions give a measure of similarity between each node and their surrounding nodes, which may be used to identify community membership. The approach is based on the concept that a node’s random walks will get trapped inside densely-connected communities. The algorithm uses random walks and we tested for the robustness of the results by setting 10 random seeds in R.

Minimum Spanning Trees (MST) were used to test sub-network structures of depressive symptoms in different groups. MST use the reduced sub-network that connects all nodes based on the identification of the minimal set of edges needed. MST provides a topological and hierarchically arranged skeleton of all nodes in the network. MSTs were estimated using the “Networktoolbox” package [7].

From the three most often used local node-specific centrality measures (i.e., closeness, betweenness and node strength), we used node strength as our primary centrality measure, because closeness and betweenness have shown to be difficult to interpret in psychological networks [8]. Strength measures the weighted number of connections of a focal node and thereby the degree to which it is involved in the network. We estimated the centrality also within potential community structures in the networks by using the centrality index called “Expected influence” that quantifies the position of a node in the network by also considering nodes that are not directly adjacent to the node. Furthermore, we computed the participation coefficient for each node, that measures the strength of a node’s connections within its community. We also calculate correlations between strength centrality measures to evaluate the overall similarity between the groups.

As additional sensitivity analyses, we performed number of analyses.

First, we bootstrapped centrality (strength and Expected influence) scores (1000 samples) to estimate the uncertainty in the correlation between the centrality scores of the DD- and DD+ groups. We then computed the correlations between each set of bootstrapped centrality scores from DD- with each set of bootstrapped centrality scores from DD+ and calculated a 95% CI as the 2.5% and 97.5% quantiles of the resulting distribution of 1,000,000 correlations following the procedure from Mullarkey and colleagues [9]. This analysis simulates the distribution of correlations we would observe if we collected 1,000 new samples of the same sizes and allows us to quantify the uncertainty around these estimates. We also evaluated the median centrality ranks of each symptom across both networks to ensure conclusion that basically the same symptoms were among the most central symptoms in both networks.

Second, we examined the community structures using the “iComDetSpin” function that extends the spinglass algorithm as it is currently implemented in the “igraph” R- package [10]<< by repeatedly performing the spinglass community detection algorithm for 5000 runs for both DD- and DD+ networks. The extended function provides (1) heatmaps with the nodes on the y- and x-axis representing the frequency across the 5000 runs with which two nodes are assigned to the same community, ordered according to their clustering (using the “pheatmap” R-package; [11] (2) all unique spinglass solutions in descending order of frequency. We reported the two most frequently occurring solutions for both DD- and DD + networks.

**References**

1. Costantini G, Richetin J, Preti E, Casini E, Epskamp S, Perugini M (2017) Stability and variability of personality networks. A tutorial on recent developments innetwork psychometrics. Personality and Individual Differences

2. Danaher P, Wang P, Witten DM (2014) The joint graphical lasso for inverse covariance estimation across multiple classes. J R Stat Soc B 76 (2):373-397. doi:10.1111/rssb.12033

3. Richetin J, Preti E, Costantini G, De Panfilis C (2017) The centrality of affective instability and identity in Borderline Personality Disorder: Evidence from network analysis. PloS one 12 (10):e0186695. doi:10.1371/journal.pone.0186695

4. Haslbeck J (2016) mgm:Estimating Time-Varying Mixed Graphical Models. R-package

5. Van Borkulo C (2015) Network Comparison Test:Permutation-Based Test of Differences in Strength of Networks. <https://github.com/cvborkulo/NetworkComparisonTest>.

6. Yang Z, Algesheimer R, Tessone CJ (2016) A Comparative Analysis of Community Detection Algorithms on Artificial Networks. Sci Rep 6:30750. doi:10.1038/srep30750

7. Christensen AP (2018) Network Toolbox: Methods and Measures for Brain, Cognitive, and Psychometric Network Analysis in R. R J 10 (2):422-439

8. Bringmann LF, Elmer T, Epskamp C, Karuse R (2018) What do centrality measures measure in psychological networks? . Preprint

9. Mullarkey MC, Marchetti I, Beevers CG (2019) Using Network Analysis to Identify Central Symptoms of Adolescent Depression. J Clin Child Adolesc Psychol 48 (4):656-668. doi:10.1080/15374416.2018.1437735

10. Csardi G, Nepusz T (2006) The igraph software package for complex network research. InterJournal, Complex Systems, 1695

11. Kolde R, Kolde MR (2015) Package ‘pheatmap. R Package, 1(7)

**Supplement Text.** Results from the sensitivity analyses

Additional sensitivity analyses showed that the correlation between strength centrality indexes between DD- and DD+ networks was 0.85 (95% 0.72, 0.94) for strength and 0.87 (95% CI 0.77, 094) for expected influence. Loss of pleasure (1), sadness (2), and loss of energy (3) had the highest median strength centrality rankings, across the 1,000 bootstrap replicates of DD- networks. In DD+ networks loss of pleasure (1), self-dislike (2) and sadness (3) had the highest median strength centrality ranking. The corresponding expected influence centrality rankings in DD- were loss of pleasure (1), sadness (2) and indecisiveness (3) and in DD+ loss of pleasure (1), self-dislike (2) and sadness (3).


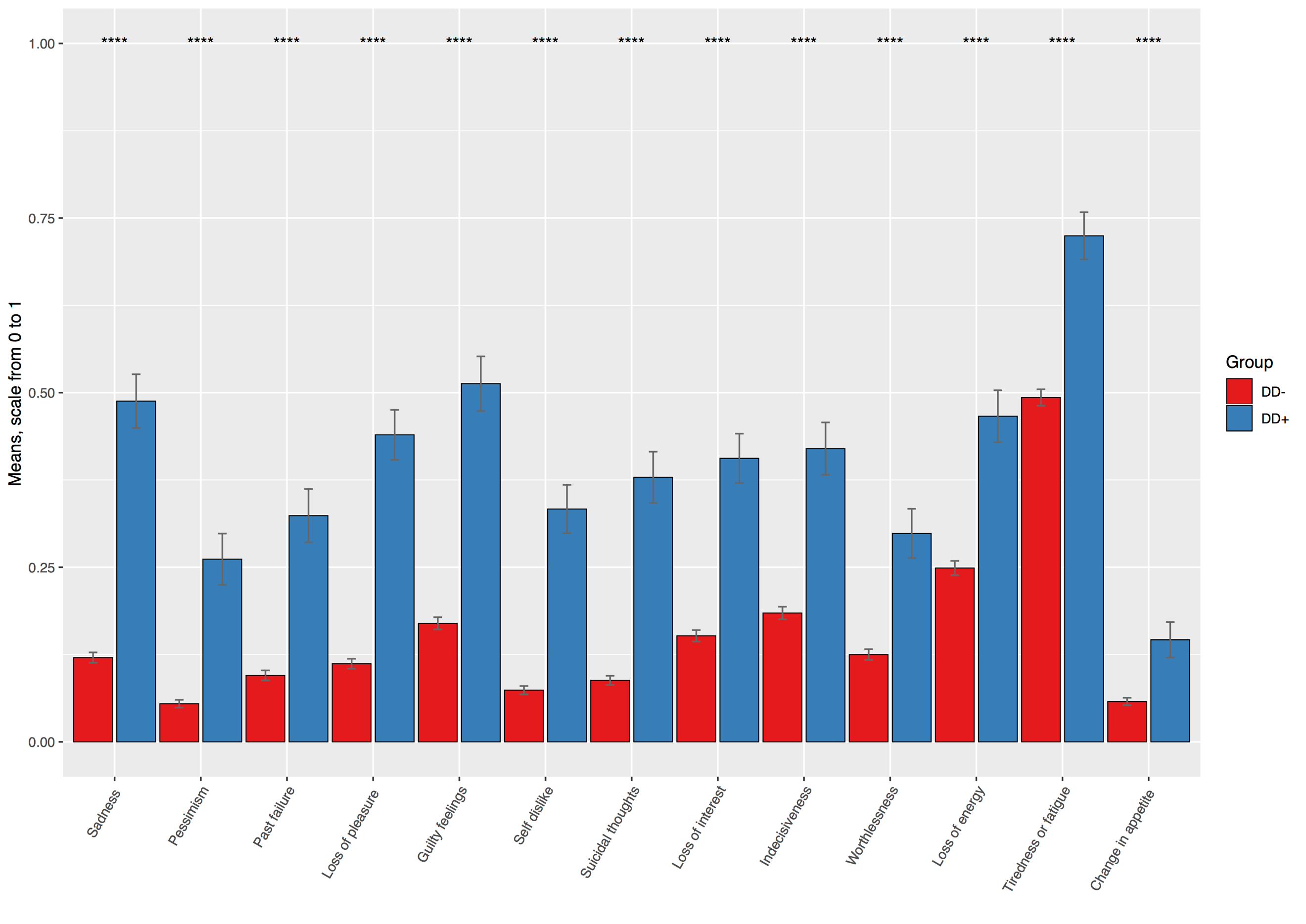


**Supplement Figure 1.** Means and SDs of the original BDI -13 – items in those without and with MDD (SDs scaled to below 1)


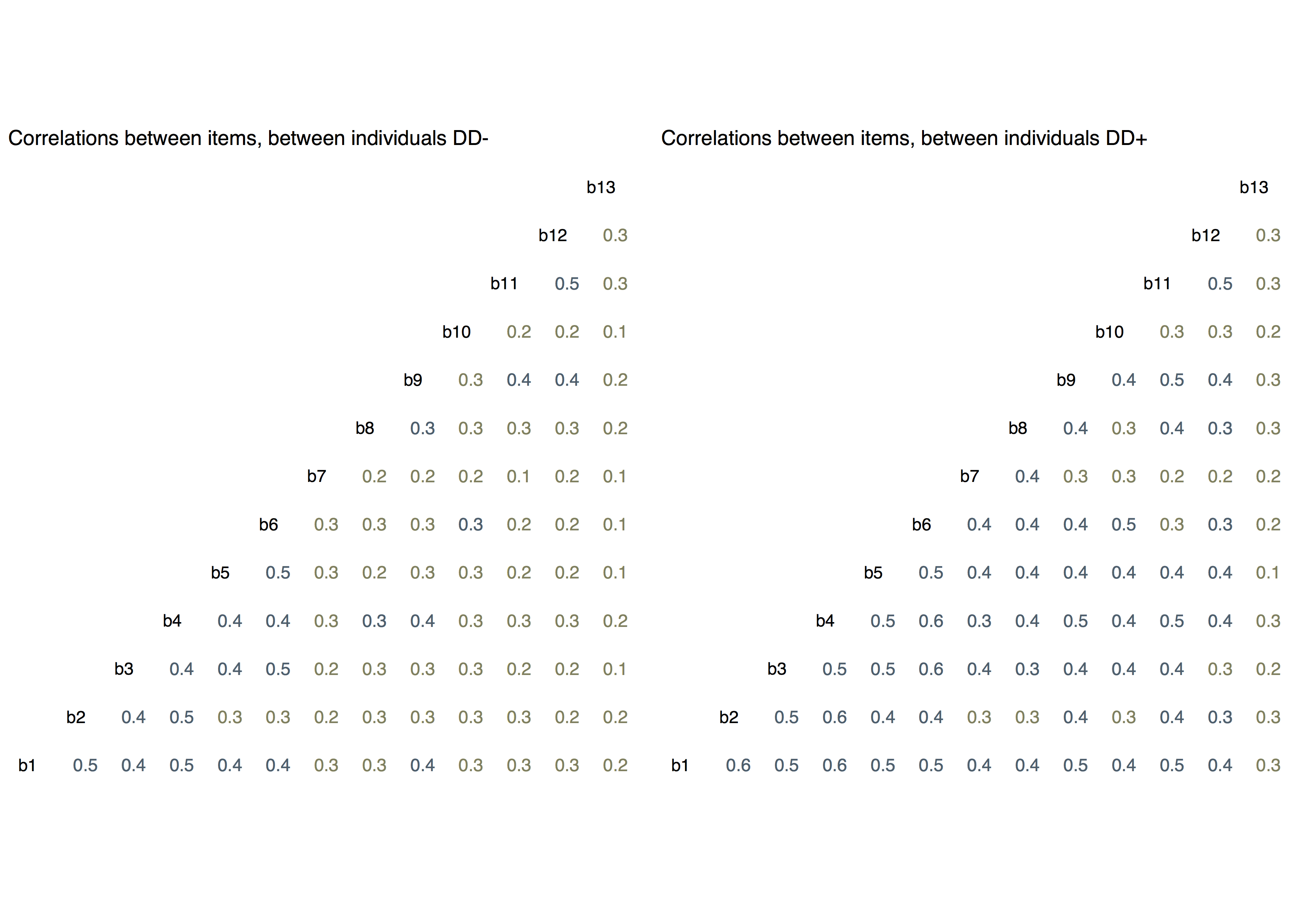


**Supplement Figure 2.** Correlations coefficients of depressive symptoms in 2000 for participants with and without MDD


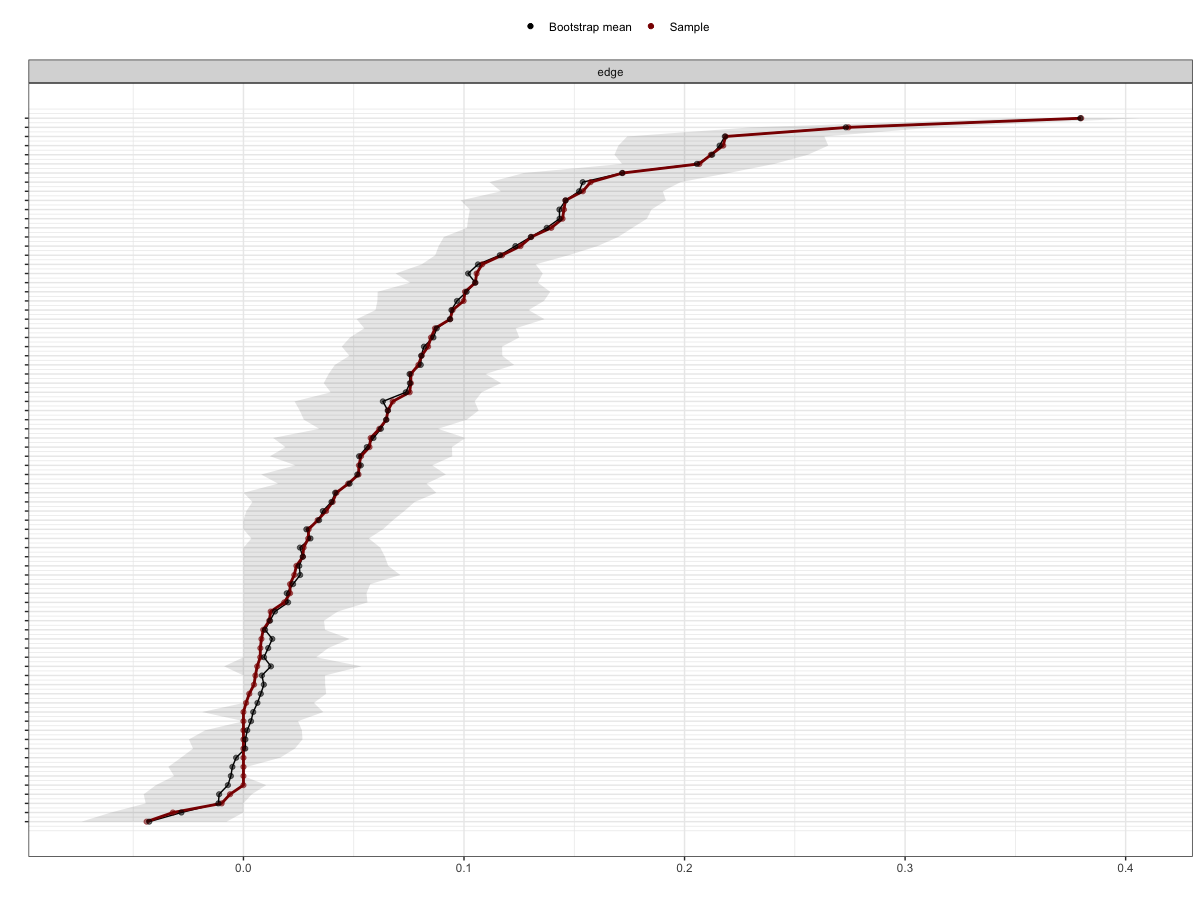

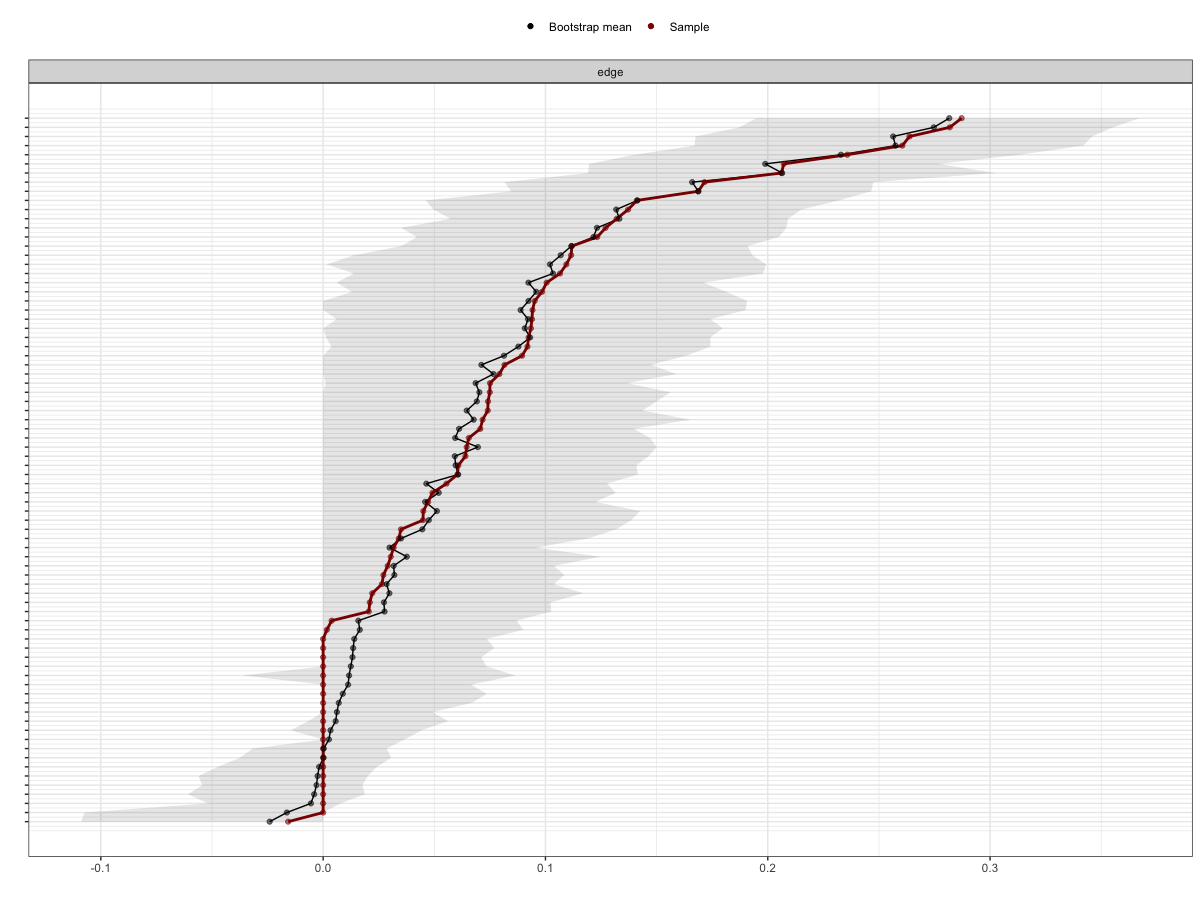


**DD - DD+**

**Supplement Figure 3.** Edge weights and Cis in DD- and DD + groups


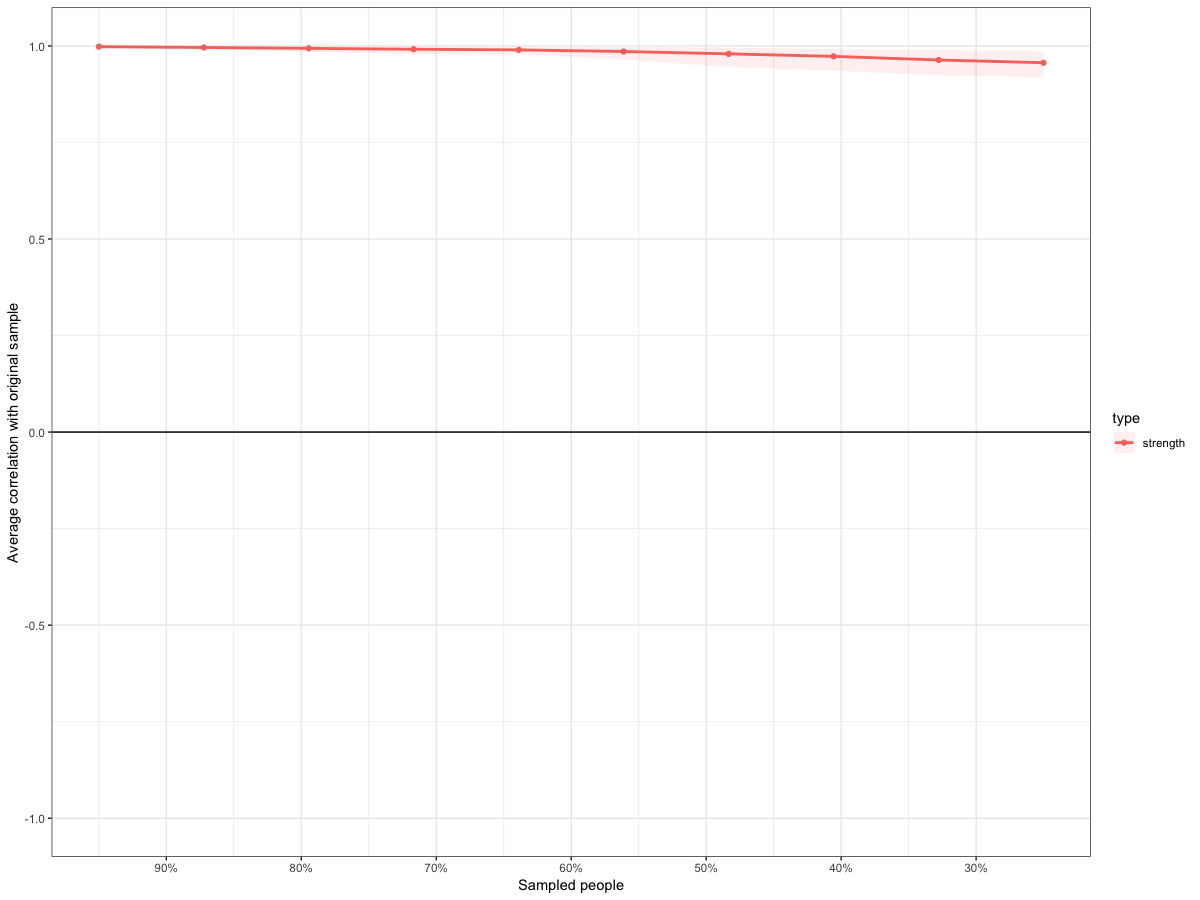

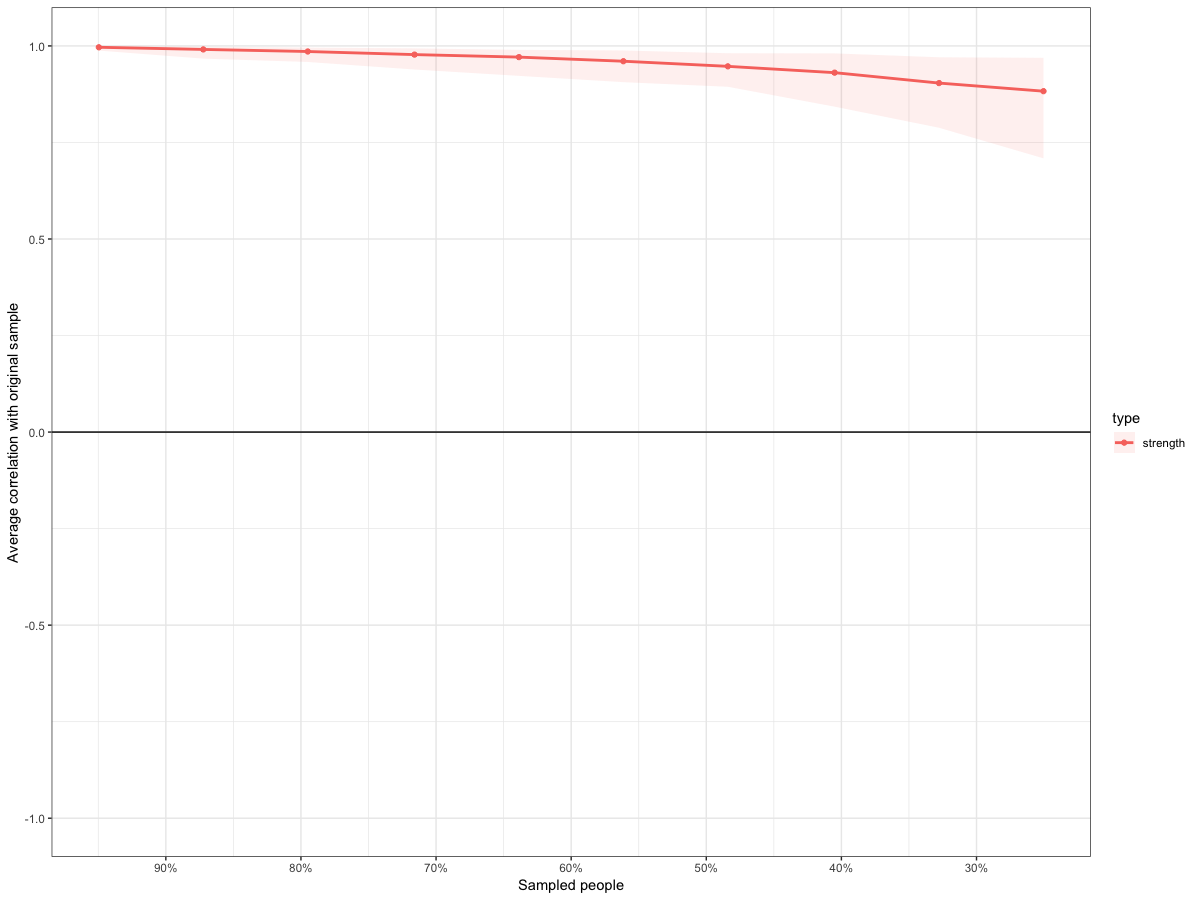


**DD- DD+**

**Supplement Figure 4.** Centrality stability in DD- and DD + groups


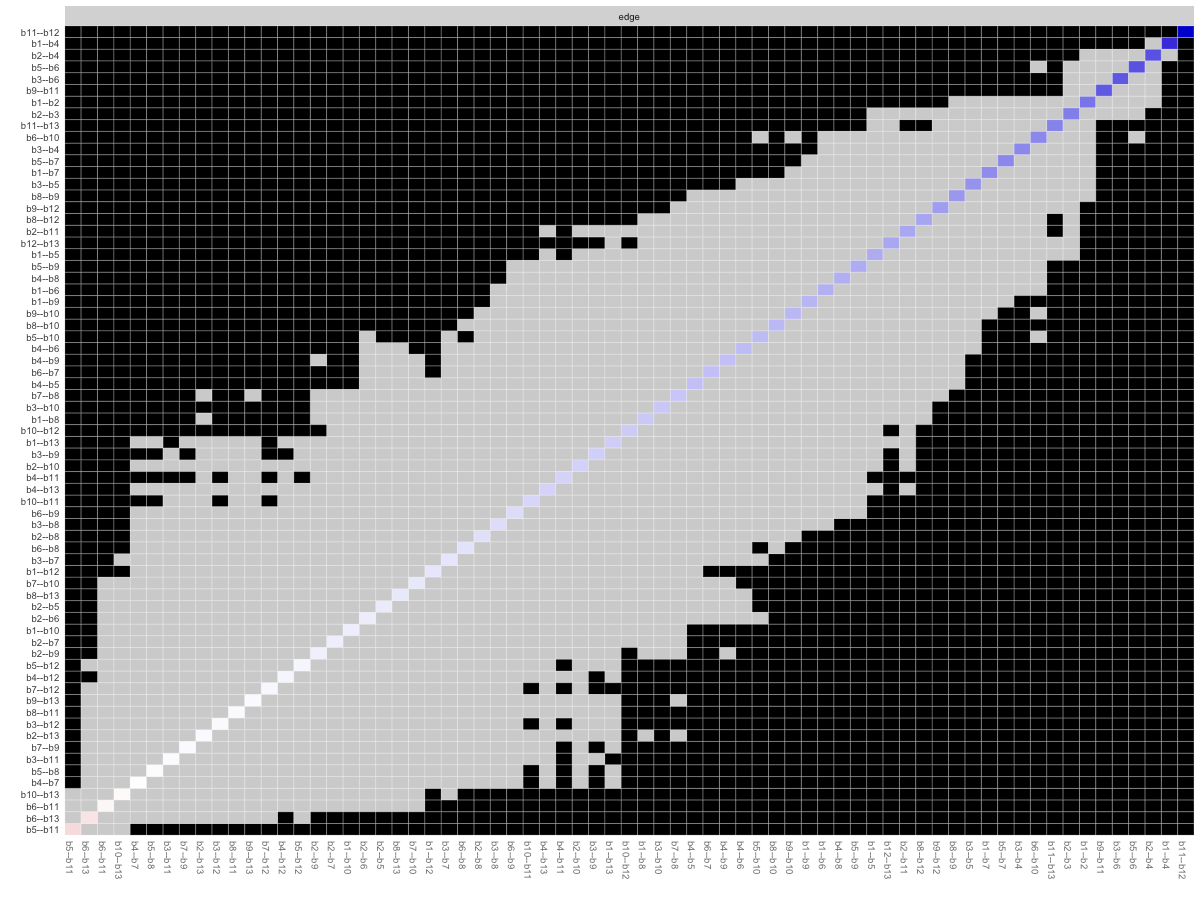


**Supplement Figure 5:** Edge weight difference test in DD- group


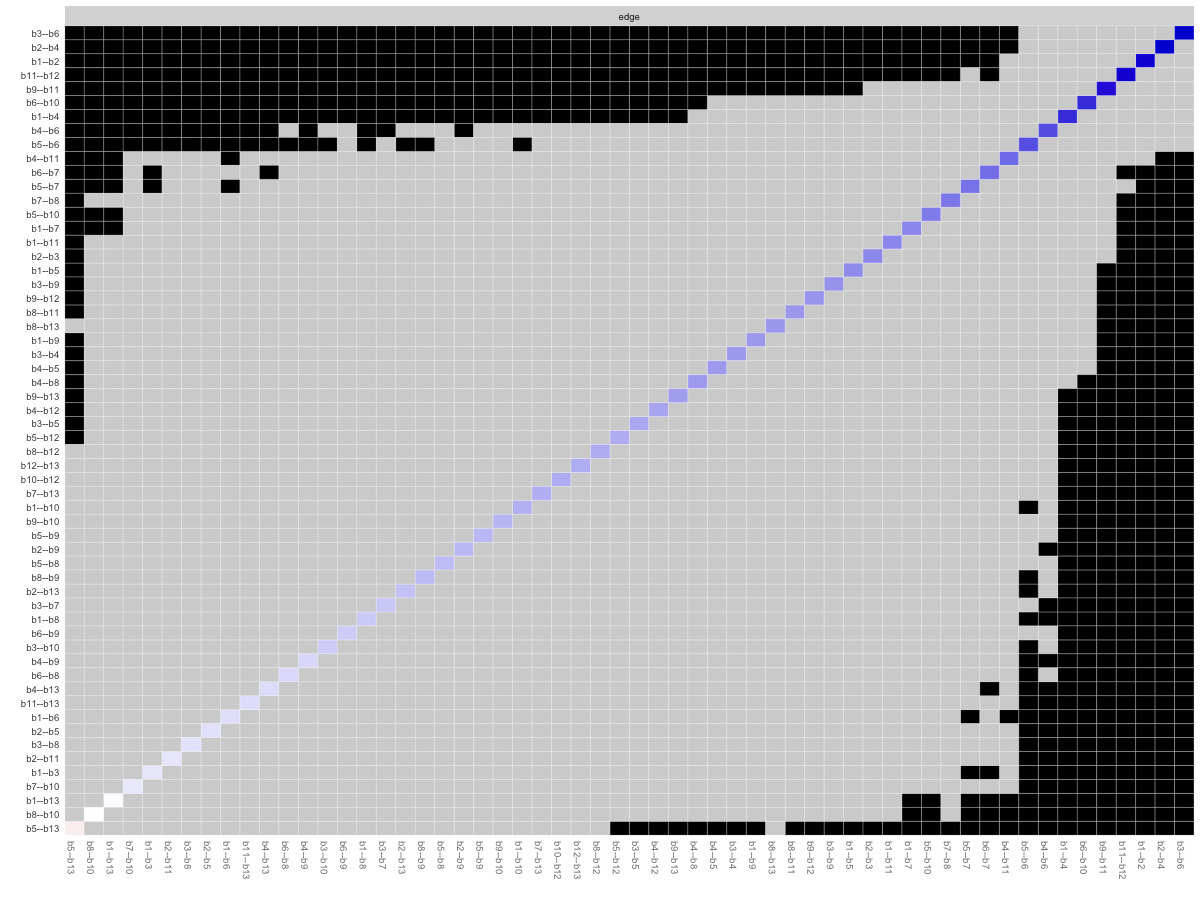


**Supplement Figure 6:** Edge weight difference test in DD+ group


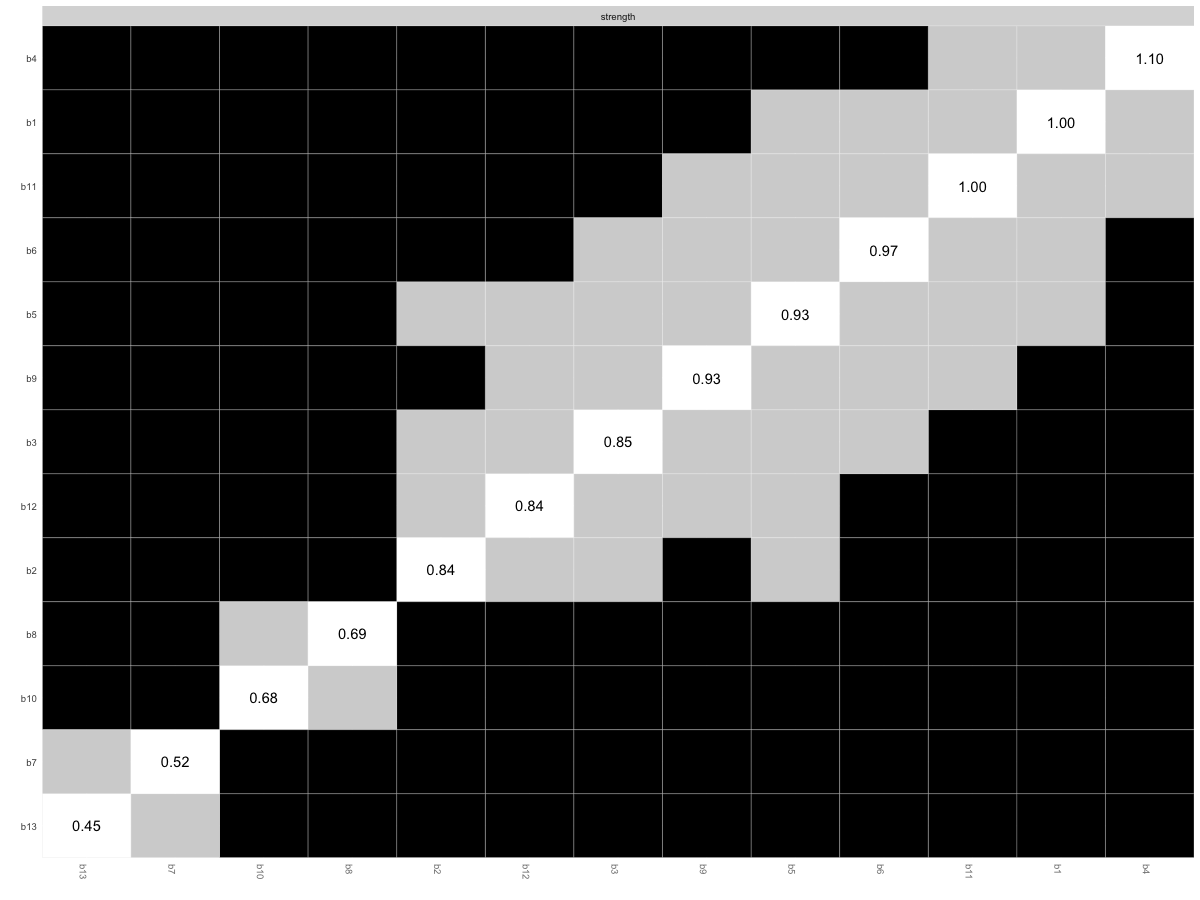


**Supplement Figure 7:** Centrality difference test in DD- group


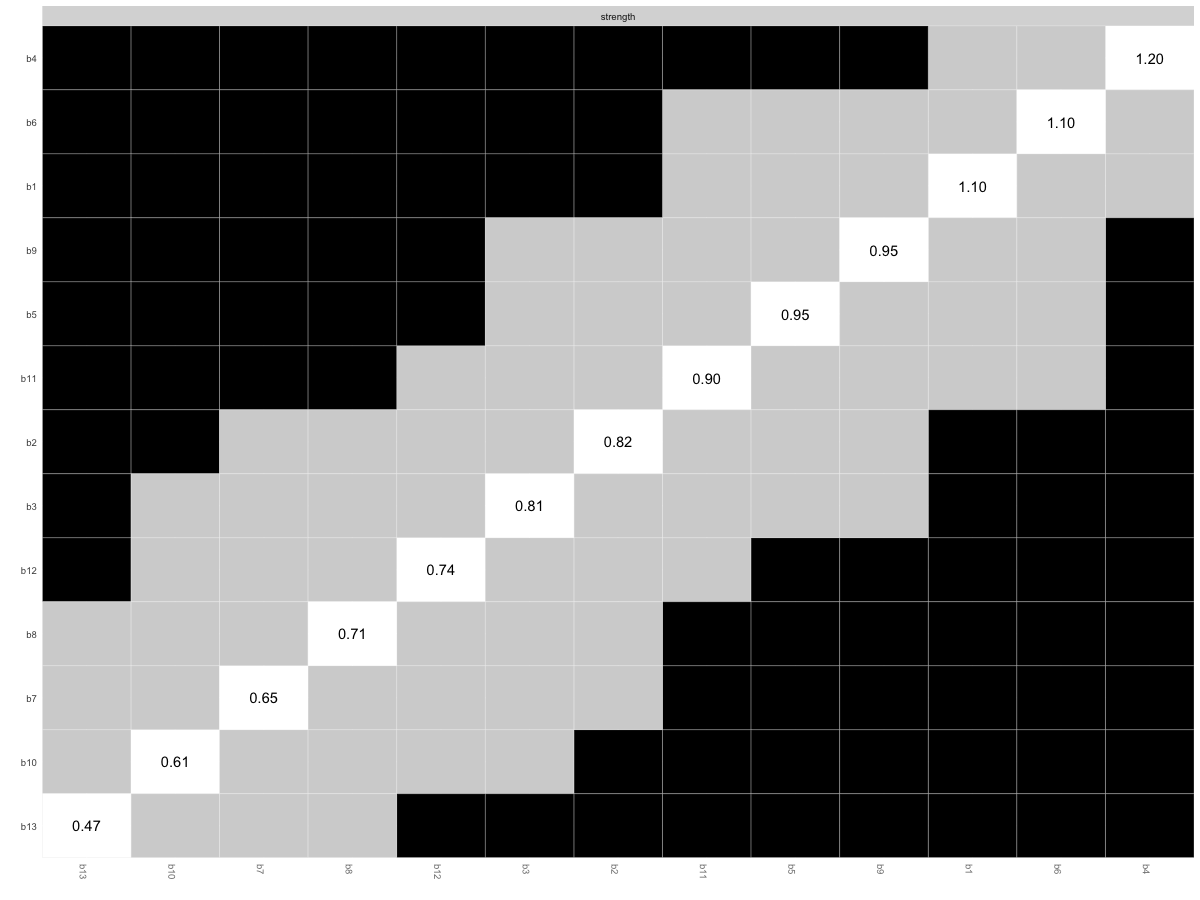


**Supplement Figure 8:** Centrality difference test in DD+ group


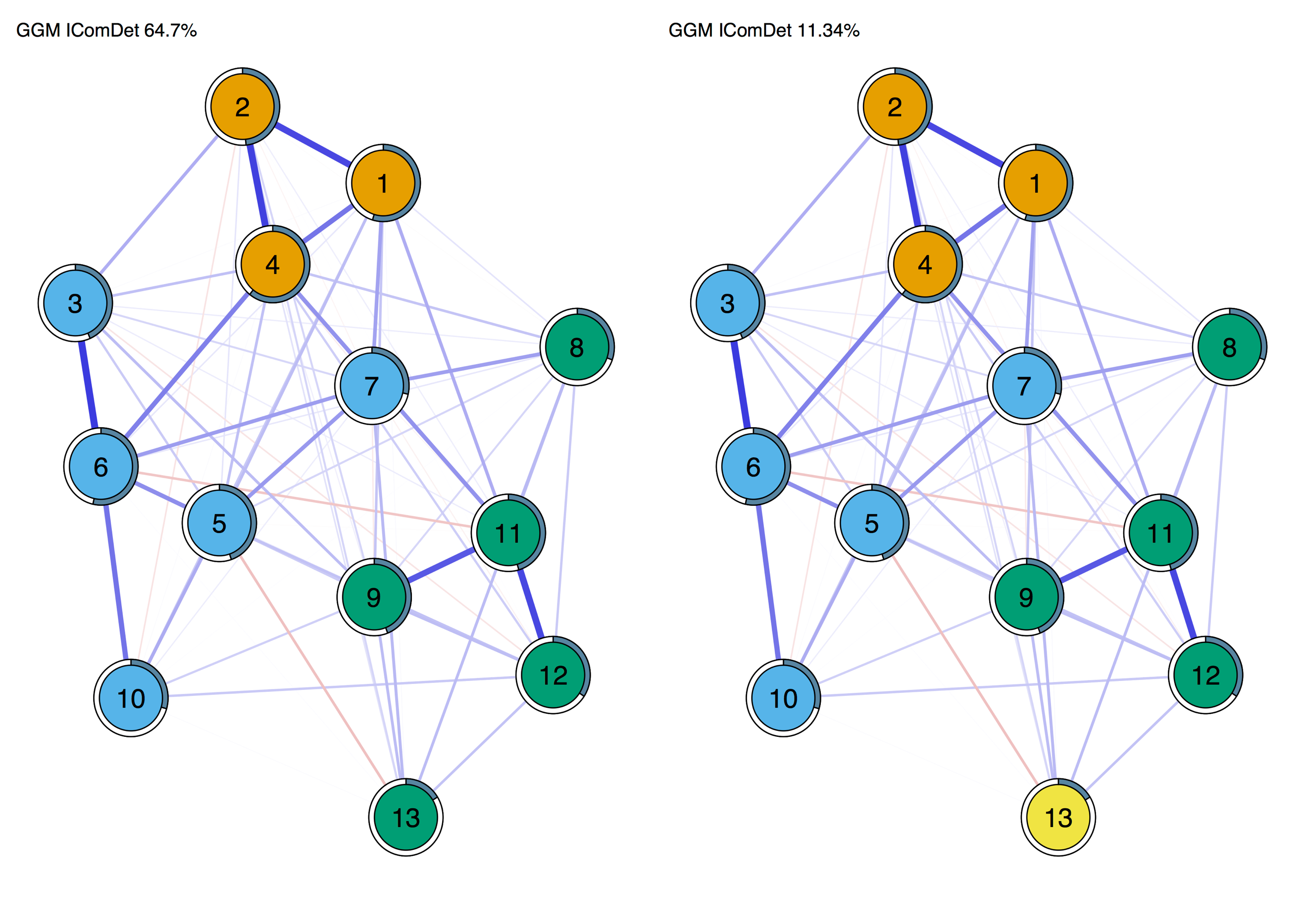


**Supplement Figure 9.** Community structures for the two most frequently occurring solutions in DD+ network


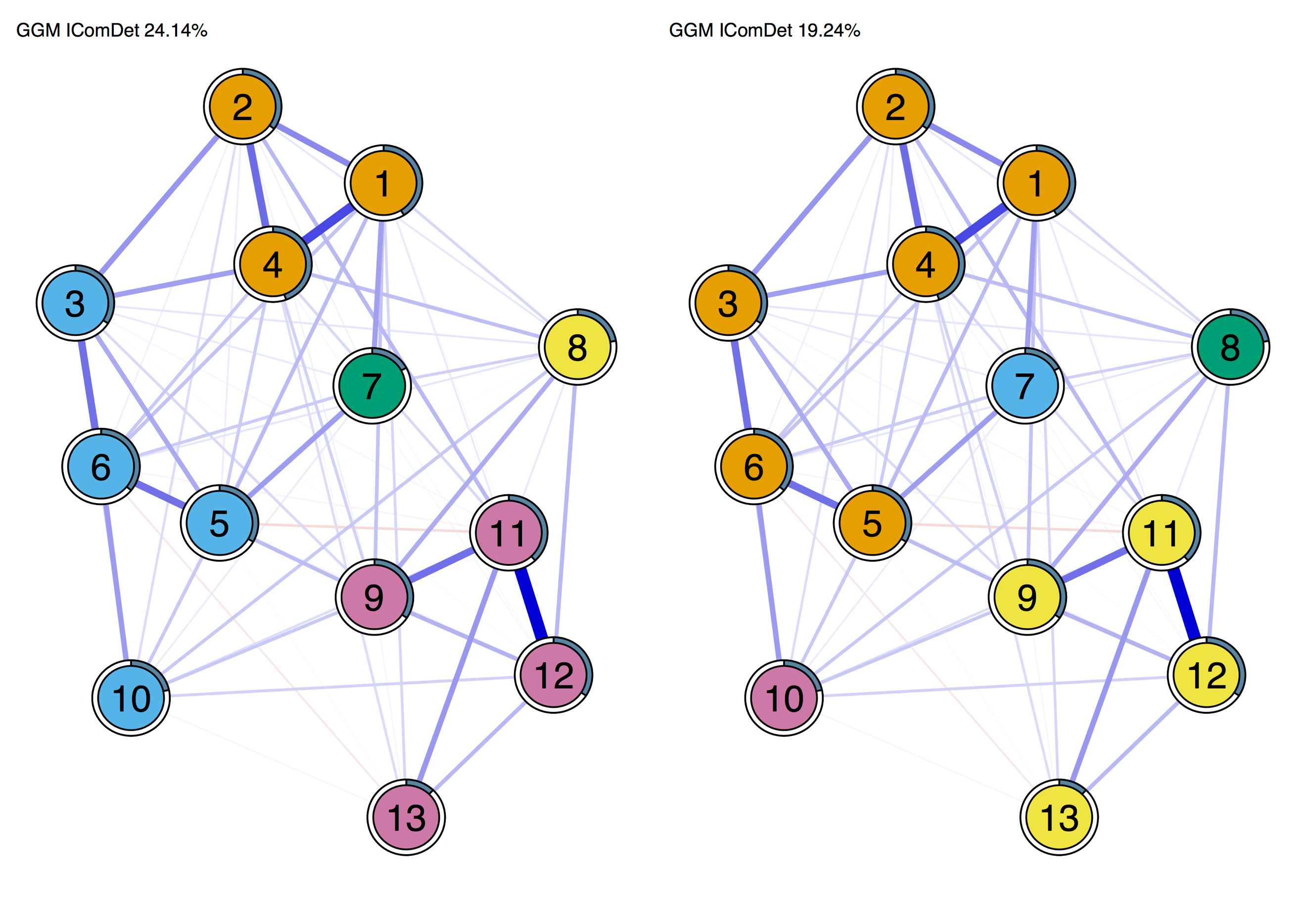


**Supplement Figure 10.** Community structures for the two most frequently occurring solutions in DD- network

**
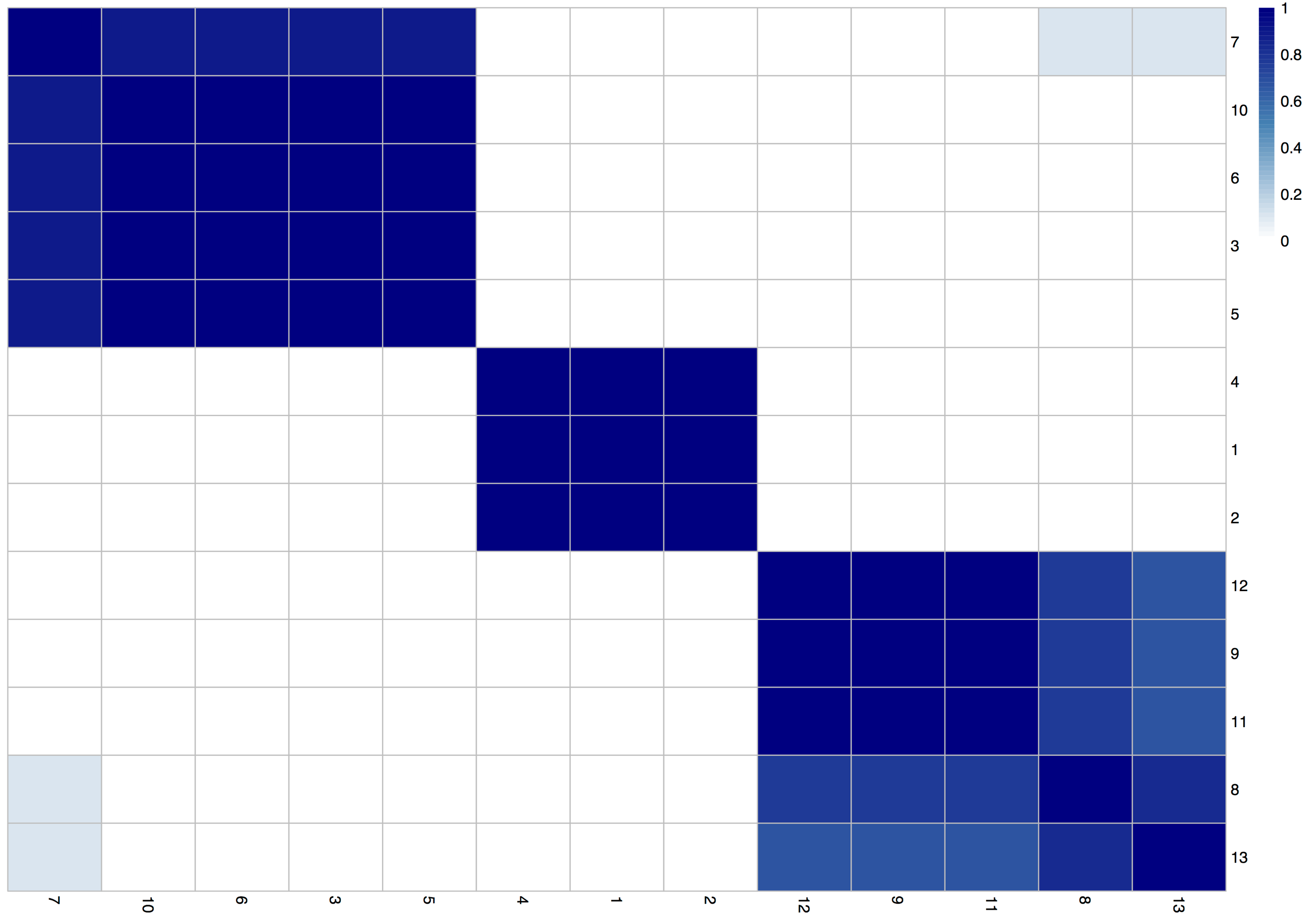
**

**Supplement Figure 11.** Heatmap for the DD+ network

**
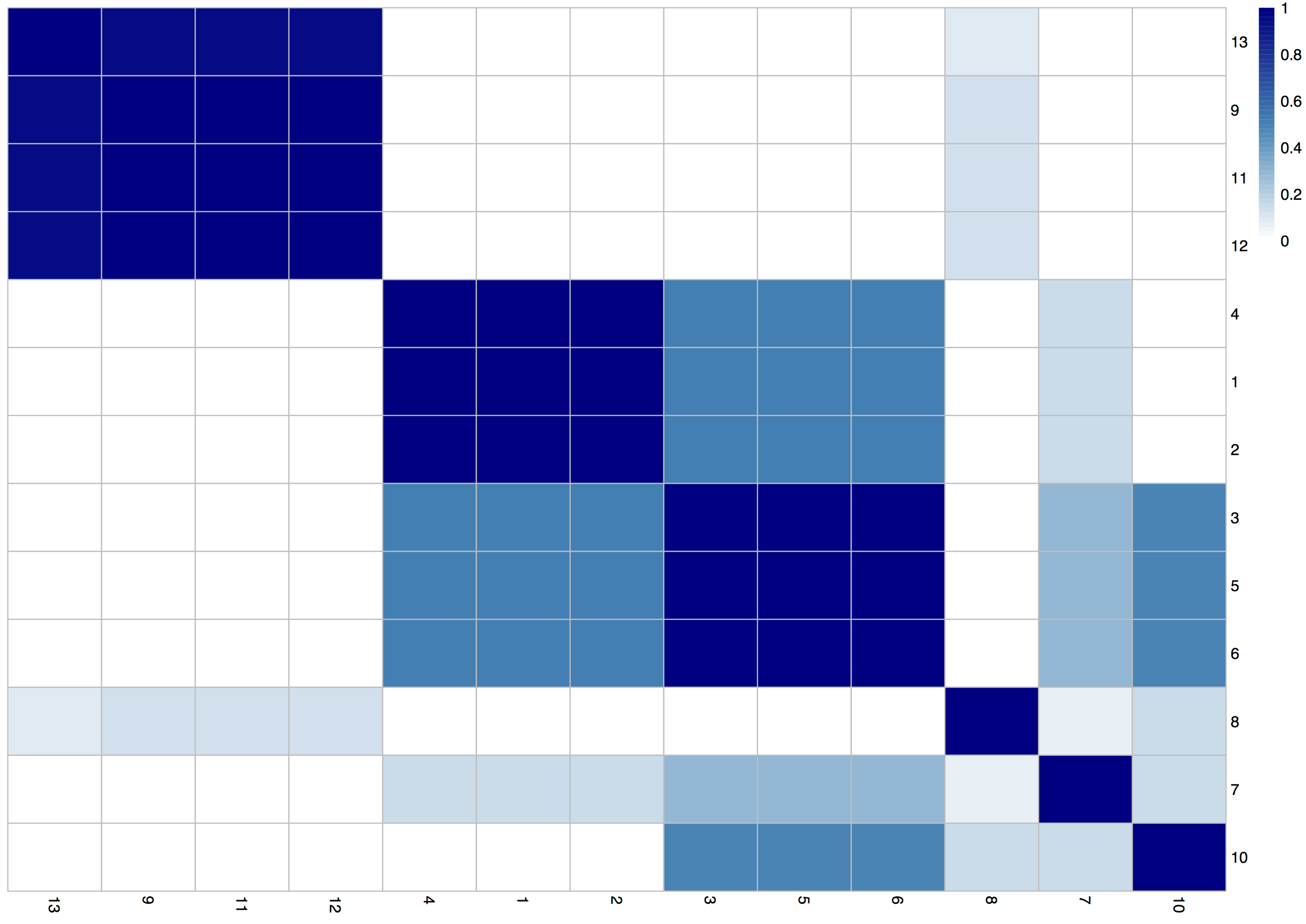
**

**Supplement Figure 12.** Heatmap for the DD- network
